# Precision RNAi for Fibrodysplasia Ossificans Progressiva: a combinatorial, unimolecular, allele selective approach

**DOI:** 10.21203/rs.3.rs-9854026/v1

**Published:** 2026-06-15

**Authors:** Yeon-Suk Yang, Katherine Y. Gross, David Cooper, Sachin Chaugule, Mi-Jeong Kim, Christopher Dahlke, Emma Mayer, Eric M. Kercher, Ji Woong Kim, Thomas Ormsby, Nozomi Yamada, Jillian Caiazzi, Samuel O. Jackson, Mohamad Omar Rachid, Dimas Echiverria, Hassan H. Fakih, Ken Yamada, Francis Y. Lee, Anastasia Khvorova, Julia F. Alterman, Jae-Hyuck Shim

**Affiliations:** 1 Department of Genetic and Cellular Medicine, UMass Chan Medical School, Worcester, MA, 01605, USA; 2 Department of Medicine, UMass Chan Medical School, Worcester, MA, 01605, USA; 3 Horae Gene Therapy Center, UMass Chan Medical School, Worcester, MA, 01605, USA; 4 RNA Therapeutics Institute, UMass Chan Medical School, Worcester, MA, 01605, USA; 5 Department of Orthopaedics and Rehabilitation, Yale University; New Haven, Connecticut, 06510, USA; 6 Department of Dermatology, UMass Chan Medical School, Worcester, MA, 01605, USA; 7 Program in Molecular Medicine, UMass Chan Medical School, Worcester, MA, 01605, USA; 8 Li Weibo Institute for Rare Diseases Research, UMass Chan Medical School, Worcester, MA, USA

## Abstract

Fibrodysplasia ossificans progressiva (FOP) is a rare genetic disorder caused by a dominant mutation in the *ACVR1* gene (R206H, 97% of cases), leading to debilitating heterotopic ossification (HO) characterized by abnormal bone growth triggered by inflammatory flare-ups.

Here, we report the development of disease-modifying, allele-selective small interfering RNA (siRNA) targeting *ACVR1*^*R206H*^. Allele selectivity is essential as wildtype ACVR1 is crucial for many functions including skeletal homeostasis and development. When conjugated to docosanoic acid (DCA), administration of the fully modified *ACVR1* siRNA, either alone or in combination with an siRNA targeting *IL1B* (a key regulator of inflammation), results in profound reduction of HO using both responsive (post-trauma) and preventative (pre-trauma) intervention strategies in a murine FOP model. Notably, the combination therapy outperforms modulation of either target alone.

We also describe the chemical engineering of a new class of lipophilic divalent siRNAs that target both pathways with a single compound, demonstrating superior muscle accumulation and therapeutic efficacy. siRNA treatment inhibits key signaling pathways (e.g. *inflammatory*, *WNT*, *Notch*, *Hedgehog*, and *TGF-β*), within muscle-resident fibroadipogenic progenitors (FAPs), leading to a significant reduction in cartilage, bone, and connective tissue formation.

This work establishes a foundation for the development of disease-modifying treatments for FOP and offers a platform for targeting other musculoskeletal disorders involving multi-pathway dysregulation.

## One Sentence Summary

Development of an allele-selective, lipophilic divalent siRNA targeting *ACVR1*^*R206H*^ and *Il1b* to suppress heterotopic ossification in a mouse model of FOP.

## Introduction

Fibrodysplasia ossificans progressiva (FOP, OMIM 135100) is an ultra-rare and severely disabling genetic disorder characterized by progressive heterotopic ossification (HO) of skeletal muscle, tendons, ligaments, fascia, and aponeuroses^1^. Initiating in early childhood and continuing into adulthood, HO follows an endochondral ossification process often triggered by flare-ups due to minor trauma, inflammation, or intramuscular injections^2^. Ectopic bone formation leads to immobility, chronic pain, and ultimately cardiorespiratory failure from thoracic insufficiency syndrome^3^. Currently, there are no definitive therapies to prevent or reverse HO; treatment is largely symptomatic, including high-dose corticosteroids during flare-ups^4^. While the FDA-approved retinoic acid receptor γ agonist palovarotene offers limited benefit, it is associated with significant side effects such as premature growth plate closure and skin toxicity^5^. Investigational agents—such as ACVR1 kinase inhibitors (IPN60130, saracatinib, INCB000928) and the anti-Activin A antibody garetosmab—are in clinical trials^6^, but concerns regarding cardiovascular safety^3,7^ and delayed fracture healing^8^ persist, underscoring the urgent need for safe, durable, disease-modifying therapies for FOP.

Approximately 97% of individuals with FOP carry a heterozygous gain-of-function mutation in the bone morphogenic protein (BMP) type I receptor *ACVR1* (c.617G>A; p.R206H)^9,10^, which enhances basal BMP signaling and confers neofunctional responsiveness to Activin A, resulting in dysregulated SMAD1/5 activation and osteo-chondrogenic differentiation^11,12^. Multiple cell types contribute to HO—including macrophages, mast cells, *Tie2*^+^ endothelial cells^13^, *Scx1*^+^ tendon progenitors^14^, and *PDGFRα*^+^*Sca1*^+^ fibroadipogenic progenitors (FAPs)^15^—with muscle-resident FAPs now recognized as key drivers of ectopic bone formation. A major therapeutic challenge lies in selectively silencing the mutant *ACVR1*^*R206H*^ allele in FAPs while preserving wildtype *ACVR1* function, which is essential for normal skeletal development, homeostasis, CNS regulation, and reproductive health^16^. A previous study has developed small interfering RNAs (siRNAs) targeting *ACVR1*^*R206H*^ or *ACVR1*^*G356D*^ allele; however, as their ability to inhibit BMP signaling in FOP patient cells was limited, further optimization is necessary to achieve therapeutic efficacy *in vivo*^17^. Additionally, *ACVR1*^*R206H*^ allele-specific artificial miRNA using recombinant adeno-associated virus-mediated delivery has demonstrated therapeutic potential in FOP mice. However, clinical application of AAV gene therapy in FOP patients requires caution, as immunological triggers associated with AAV delivery may increase the risk of HO induction^18,19^. In addition to dysregulated BMP signaling, inflammation, particularly through interleukin-1 beta (IL-1B), is a key driver of flare-up-associated HO in FOP^20^. IL-1B levels increase during flare-ups and correlate with new lesion formation, promoting a pro-inflammatory environment that supports fibroproliferation, chondrogenesis, and osteogenesis^21,22^. Secreted by macrophages and infiltrating immune cells, IL-1B has emerged as a therapeutic target, with case reports showing that IL-1 blockade—using canakinumab or anakinra—can reduce flare frequency and limit HO progression in patients with FOP^23^. Currently, several clinical trials are underway globally to investigate the off-label use of anti-IL1 therapies for FOP treatment (ClinicalTrials.gov: NCT06724562). Together, aberrant ACVR1 signaling and IL-1–mediated inflammation constitute two interdependent pathogenic axes in FOP, and their combined targeting may provide more comprehensive disease control than approaches targeting a single pathway.

siRNAs are a promising class of RNA therapeutics capable of treating a broad range of genetic diseases, provided effective tissue-specific delivery^24,25^. To date, seven siRNA drugs have been approved^26^, all targeting the liver most with multivalent GalNAc conjugation, enabling efficient hepatocyte uptake and durable gene silencing lasting 6–12 months^27^. Following uptake, chemically stabilized siRNAs are retained in endosomes and lysosomes, which serve as intracellular depots for gradual cytoplasmic release^28,29^. Their sequence specificity, prolonged activity, and ability to modulate multiple pathways with infrequent dosing make siRNAs especially attractive for chronic, multisystemic conditions like FOP. We previously demonstrated that increasing molecular valency^30,31^ and incorporating lipophilic conjugates^32,33^ significantly enhance both local and systemic siRNA delivery. In particular, conjugation to docosanoic acid (DCA) enables efficient, safe delivery to extrahepatic tissues, including muscle^32,34^, and is currently under evaluation in phase II clinical trials targeting *sFLT1* and *JAK1* (ClinicalTrials.gov: NCT05881993, NCT06826196).

Here, we report the chemical engineering and *in vitro* and *in vivo* validation of allele-selective siRNAs targeting *ACVR1*^*R206H*^. In patient-derived induced pluripotent stem (iPS) cells, treatment with these siRNAs shifted mutant:wild-type mRNA allele ratio from ~80:20 to 3:97. Furthermore, in healthy *ex vivo* human muscle tissue lacking the R206H mutation, the potency of siRNA targeting *ACVR1*^*R206H*^ was markedly reduced, suggesting potent and selective silencing of the pathogenic transcript. Given the central role of inflammation, we also screened and identified a chemically stabilized siRNA targeting *Il1b* with robust efficacy and drug-like properties. We show that lipophilic conjugation of these siRNAs leads to efficient muscle and HO delivery, target silencing and significant reduction in HO both in therapeutic and prophylactic FOP models. Interestingly, *ACVR1*^*R206H*^ and *Il1b* targeting alone provides measurable benefit, while combinatorial treatment with *ACVR1*^*R206H*^ and *Il1b* siRNAs results in superior disease control. Finally, we introduce a unimolecular divalent siRNA capable of simultaneously silencing *ACVR1*^*R206H*^ and *Il1b*. This dual-targeting molecule achieves effective delivery to both FAPs and immune cells, blocking multiple osteo-chondrogenic and inflammatory signaling pathways, and preventing HO formation in FOP. Remarkably, increasing the size of these lipophilic divalent siRNAs enhances tissue accumulation and efficacy, establishing a proof-of-concept for a modular, combinatorial siRNA platform for the treatment of FOP and other multi-pathway musculoskeletal disorders.

## Results

### Development of an ACVR1^R206H^ allele-specific siRNA

**Figure 1.**
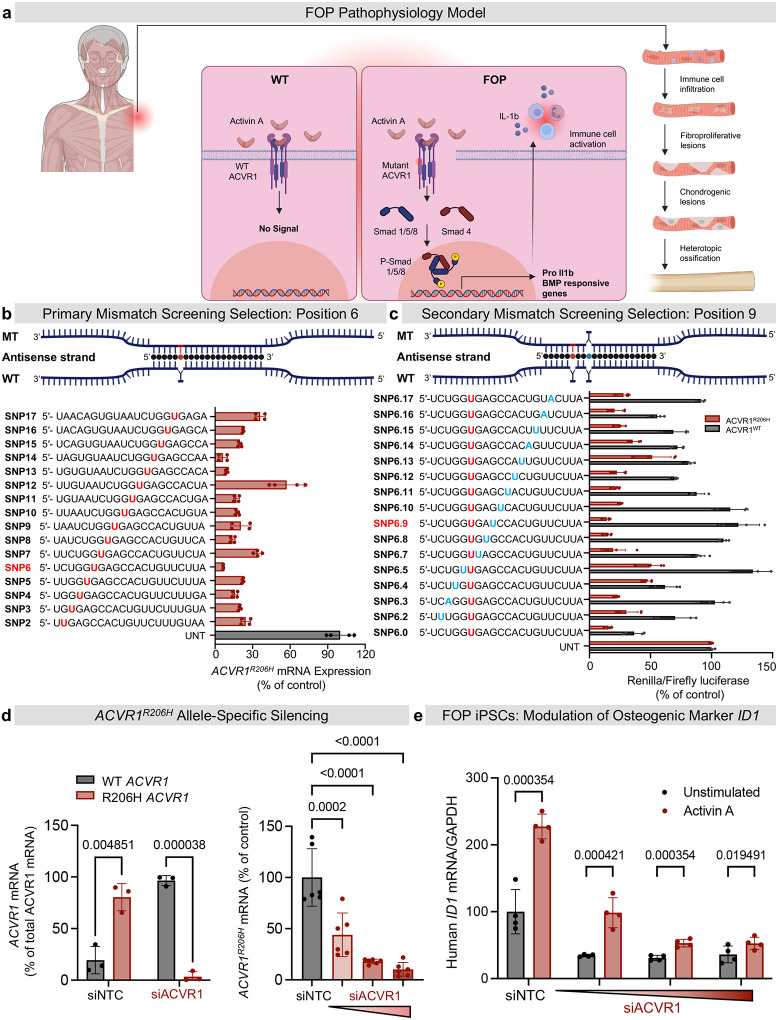
Development of human *ACVR1*^*R206H*^ allele-specific siRNA. **a.** Schematic diagram showing the molecular and cellular mechanisms of FOP pathogenesis (created with biorender.com). **b, c.** Schematic diagram showing SNP screening strategy, detailing potential on-target (*ACVR1*^*R206H*^ allele) and off-target (*ACVR1*^*WT*^ allele) complementarity with primary (**b**) and secondary (**c)** mismatches. A panel of 16 siRNAs was designed by walking the 21-nucleotide antisense strand sequence around the c.617>A SNP site. *ACVR1*^*R206H*^ BMSCs were treated with each siRNA at 1.5 μM and 3 days later, *ACVR1*^*R206H*^ mRNA levels were assessed by RT-PCR as a percentage of untreated (UNT, n = 4, **b**). A panel of 16 siRNAs derived from sequence SNP6 were generated with a secondary mismatch along the antisense strand, and their on-target and off-target activities were assessed using the reporter plasmid that contains firefly luciferase (Fluc), Renilla luciferase (RLuc), with complementary sequences of human *ACVR1*^*R206H*^ or *ACVR1*^*WT*^ incorporated into the 3’ UTR of RLuc. 1 day after siRNA treatment, HEK293 cells were transiently transfected with the reporter plasmid. 2 days later, a luciferase assay was performed to measure *Renilla* luciferase and normalized to firefly luciferase. Lower activities indicate higher silencing efficacy of siRNAs (n = 3, **c**). **d.** Human FOP iPSCs were treated with 1.0 μM siACVR1 or siNTC, cultured under osteogenic conditions for 4 days, and subjected to next-generation sequencing (NGS) for transcript quantification: *ACVR1*^*R206H*^ vs*. ACVR1*^*WT*^ (n = 3, **left**). Additionally, a dose-dependent assay with siACVR1 was performed in *ACVR1*^*R206H*^ BMSCs to evaluate silencing efficiency by RT-PCR (*n* = 6, **right**). **f.** Human FOP iPSCs were treated with siACVR1 in a 3-point dose response. After 72 hours, cells were treated with Activin A (50 ng/ml) for 12 hours, and *Id1* expression was assessed by RT-PCR (n = 4). Data are representative of three independent experiments. Values represent mean ± SD by one-way ANOVA test **(b-e)**.

Given that a heterozygous activating mutation of *ACVR1* (c.617G>A;p.R206H) accounts for approximately 97% of FOP patient populations^10^, we hypothesized that selectively inhibiting the ACVR1^R206H^ receptor—without impacting the wildtype ACVR1^WT^ receptor—would ablate aberrant BMP signaling responsible for HO in the patients of FOP (Fig. 1a). Building on our previous work employing a thermodynamic compensation approach to optimize fully chemically modified siRNAs^35^, we designed a panel of sixteen chemically stabilized siRNAs overlapping the single nucleotide polymorphism (SNP) of human *ACVR1*^*R206H*^ mRNA to achieve allele-specific silencing. The sequences and chemical modification patterns of these siRNAs are detailed in **Extended Table 1**. The siRNAs incorporate 2’-O-methyl (2’-OMe) and 2’-deoxy-2’-fluoro (2’-F) ribose modifications, with terminal backbones modified with phosphorothioates (PS).

Primary screening in mouse bone marrow-derived stromal cells expressing human *ACVR1*^*R206H*^ receptor (*ACVR1*^*R206H*^ BMSCs) identified eight siRNA sequences (SNP3, 6, 8, 9, 10, 11, 13, 14) that achieved 80–90% silencing efficiency of *ACVR1*^*R206H*^ mRNA compared to untreated (Fig. 1b). Among them, five siRNA sequences (SNP3, 6, 8, 9, 14) significantly reduced BMP-responsive gene *Msx2* after Activin A stimulation—consistent with *ACVR1*^*R206H*^ silencing (**Extended Fig. 1a–b**). However, in FOP patient-derived induced pluripotent stem cells (iPSCs), both *ACVR1*^*R206H*^ and *ACVR1*^*WT*^ mRNA levels were decreased following siRNA treatment, indicating limited discrimination of the heterozygous SNPs (**Extended Fig. 1c**). To further assess on-target versus off-target effects, we generated two reporter plasmids containing firefly luciferase (Fluc) and *Renilla* luciferase (RLuc) with complimentary sequences of human *ACVR1*^*R206H*^ and *ACVR1*^*WT*^ in the 3’-UTR of RLuc (**Extended Fig. 2a**). RLuc activity was normalized to FLuc activity, with lower luciferase activity indicating higher silencing efficiency. As observed in human FOP iPSCs, siRNA treatment reduced both *ACVR1*^*R206H*^ and *ACVR1*^*WT*^ luciferase activities in a dose-dependent manner, with little discrimination between the two, suggesting that a single mismatch is insufficient for allele-specific discrimination of *ACVR1* SNP heterozygosity (**Extended Fig. 2b**).

Since a second mismatch can substantially reduce the guide strand’s affinity for the non-targeted isoform^35^, we introduced secondary mismatches into the two top performing siRNAs from the primary screen, SNP6 and SNP8, to enhance allele specific discrimination. The sequences and chemical modification patterns of these siRNAs are detailed in **Extended Table 2**. The addition of secondary mismatches in SNP6 at positions 7, 8, 9, and 11 significantly improved the target/non-target discrimination (Fig. 1c). To quantify these differences, a 7-point dose-response assay revealed that a secondary mismatch at position 9 provided the best discrimination when the primary mismatch was at position 6. This optimized siRNA is herein referred to as siACVR1 (siACVR1 MT IC50: 6.23 nM, siACVR1 WT IC50: 23.6, **Extended Fig. 2c–d**).

Due to the difficulty in obtaining large numbers of cells from FOP patients—given the substantial risk of inducing HO—human FOP iPSCs were used to evaluate whether siACVR1 could discriminate heterozygous SNPs in a native context. Sanger sequencing demonstrated evidence of siACVR1 selectivity for *ACVR1*^*R206H*^ mRNA in these cells (**Extended Fig. 3a**). This is consistent with next generation sequencing (NGS) analysis which showed that while untreated FOP cells exhibited 80.58% (~609 transcripts) *ACVR1*^*R206H*^ and 19.42% (~130 transcripts) *ACVR1*^*WT*^ transcripts, treatment with siACVR1 drastically shifted the expression to 3.46% (~23 transcripts) *ACVR1*^*R206H*^ and 96.52% (~539 transcripts) *ACVR1*^*WT*^ (Fig. 1d, **left, Extended Fig. 3b**). Human WT BMSCs were cultured under osteogenic conditions following siRNA treatment. In these cells, siACVR1 treatment did not alter mRNA levels of *ACVR1*^*WT*^ and osteogenic marker genes, including osteocalcin (*BGLAP*) and bone sialoprotein (*IBSP)*, indicating little to no effects of siACVR1 on endogenous *ACVR1*^*WT*^ expression and osteogenesis in human WT cells (**Extended Fig. 3c–d**). By contrast, siACVR1 treatment of human FOP cells attenuated the induction of the BMP-responsive gene *ID1* following Activin A stimulation (Fig. 1e). These results suggest that the mutant targeting siACVR1 significantly impacts the ratio of mutant to wild-type *ACVR1* mRNA and inhibits BMP signaling associated with FOP by reducing the interaction of the ACVR1^R206H^ receptor with Activin A.

To examine the ability of siACVR1 to inhibit osteogenic differentiation of FOP cells *in vitro*, human *ACVR1*^*R206H*^-expressing BMSCs were isolated from 4-week-old *ACVR1*^*(R206H)Fl*^*;Prrx1-cre* mice (hereinafter referred to as *ACVR1*^*R206H*^ BMSCs) and treated with different doses of siACVR1 under osteogenic conditions. This treatment markedly reduced expression of *ACVR1*^*R206H*^ (Figure 1d, **right**), but did not alter mRNA levels of mouse *Acvr1* (**Extended Figure 3e**). Reduction in *ACVR1*^*R206H*^ mRNA levels corresponds to a dose dependent decrease in expression of osteogenic marker genes, *Bglap*, type 1 collagen (*Col1a1*), and *Ibsp* (**Extended Fig. 3f**) and alkaline phosphatase activity (ALP), an early osteogenic marker (**Extended Fig. 3g**). The *ACVR1*^*R206H*^ mutation is known to enable aberrant activation of SMAD1/5-mediated BMP signaling in response to Activin A, promoting osteogenic differentiation^11,12,36^. As expected, Activin A treatment significantly upregulated *Id1* expression and SMAD1/5 phosphorylation in untreated *ACVR1*^*R206H*^ BMSCs (**Extended Fig. 4a–b**); however, both effects were reduced by siACVR1 treatment in a dose-dependent manner. Activin A-induced osteogenic differentiation was diminished in the presence of siACVR1, as evidenced by lower ALP activity and extracellular mineralization (**Extended Fig. 4c–d**). Similarly, siACVR1 treatment attenuated Activin A-induced glycosaminoglycan extracellular production (**Extended Fig. 4e**) and chondrogenic gene expression (**Extended Fig. 4f**). These findings demonstrate that siACVR1 inhibits Activin A-driven aberrant BMP signaling, osteogenesis, and chondrogenesis of FOP cells. Of note, TGFβ- and WNT3a-responsive transcription activities, as well as FGF2-induced phosphorylation of ERK MAPKs, were normal in siACVR1-treated cells (**Extended Fig. 5**), indicating that siACVR1 specifically inhibits Activin A signaling without affecting signaling by other osteogenic ligands, such as TGFβ, WNTs, and FGFs.

### DCA-conjugated siACVR1 is a muscle-targeting suppressor of HO in FOP

**Figure 2.**
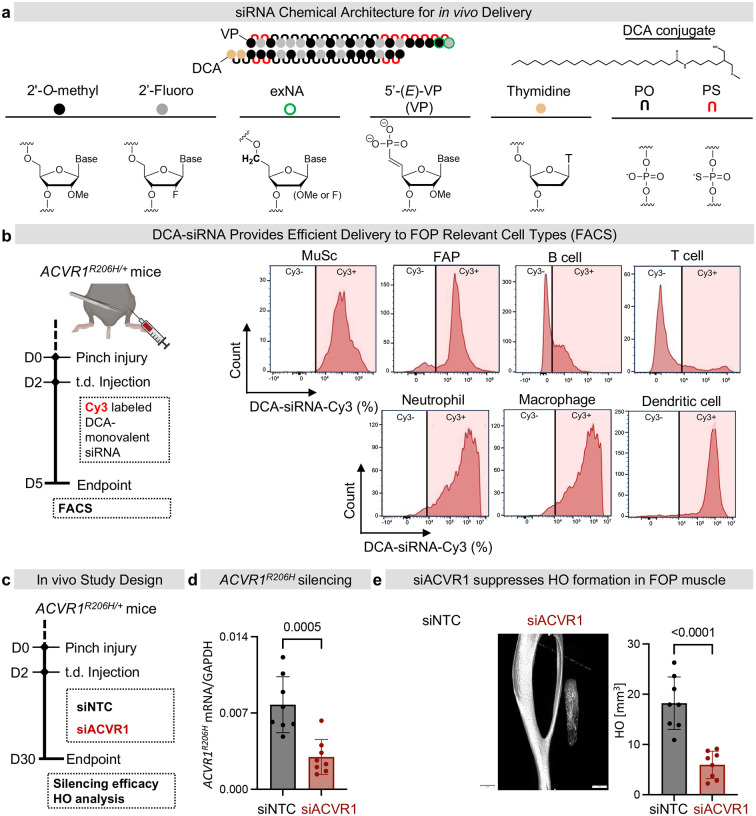
Local delivery of DCA-conjugated siRNA targeting *ACVR1*^*R206H*^ suppresses HO in FOP mice. **a.** Chemical modifications applied along the siRNA backbone to enhance stability, including 2’-O-methyl (2’-OMe), 2’-fluoro (2’-F), 5’-vinyl phosphonate (5’-(E)-VP), phosphodiester (PO), phosphorothioate (PS), and extended nucleic acid (exNA). **b.** 10 nmol DCA monovalent siRNA was injected t.d. into the tibial muscle of 8-week-old *ACVR1*^*R206H/+*^ mice 2 day after pinch injury. 3 days later, cells were isolated from digested muscle near the site of injury and subjected for FACS analysis to assess the frequency of the indicated cell types. MuSc: muscle satellite cells, FAP: fibroadipogenic progenitor. **c-e.** Schematic diagram showing study design (**c**). A single dose of siNTC or siACVR1 (10 nmol) was injected t.d. into the tibial muscle of 8-week-old *ACVR1*^*R206H/+*^ mice 2 days after pinch injury. 4 weeks later, *ACVR1*^*R206H*^ expression in injure muscle was measured by RT-PCR (n = 7, **d**), and HO was assessed by microCT imaging and quantification (n = 8, **e**). Scale bars: 5 mm. Data are representative of two independent experiments. Values represent mean ± SD, with statistical significance determined by two-sided unpaired t-test **(d, e)**.

It has previously been shown that docosanoic acid (DCA) conjugation to siRNA enables efficient, sustained, and non-toxic gene silencing in skeletal muscle^34^. Therefore, we synthesized DCA-conjugated, fully chemically modified siRNAs with a balanced content of 56% 2′-O-methyl (2′-O-Me) and 44% 2′-Fluoro (2′-F) sugar modifications, phosphorothioate tails, and an extended nucleic acid (exNA) modification near the 3′ end of the guide strand to significantly increase exonuclease resistance^37^ (Fig. 2a). These siRNAs were used to investigate their efficacy in a mouse model of FOP harboring the heterozygous human *ACVR1*^*R206H*^ allele (*ACVR1*^*R206H/+*^). Due to the perinatal lethality of constitutively expressed human *ACVR1*^*R206H*^ allele^38^, FOP mice were generated by crossing mice with a conditional knock-in allele of human *ACVR1*^*R206H*^ (*ACVR1*^*(R206H)Fl*^) and Sox2-Cre mice, where Cre recombinase expression in epiblasts drives *ACVR1*^*R206H*^ expression in all tissues. Since intramuscular injection often induces trauma that can trigger HO formation in FOP patients^39^, all studies for local delivery of siRNAs were performed with a hollow microneedle^40^ for transdermal (t.d.) injection to minimize muscle trauma and its potential to provoke HO.

Previous studies have reported that traumatic injury induces sequential pathological changes in the skeletal muscle of FOP mice during HO progression, including immune cell infiltration (Day 1–3), muscle degeneration and fibroproliferation (Day 3–7), chondrogenesis (Day 7–14), and osteogenesis with heterotopic bone marrow formation (Day 14–28)^13,41,42^. Building on this understanding, we assessed whether post-injury treatment with siACVR1 could mitigate injury-induced HO, potentially offering a therapeutic window to prevent HO after trauma occurs. First, to identify the cell populations targeted by DCA-conjugated siRNA in injured muscle of FOP mice, PBS or DCA-conjugated siRNA labeled with Cy3 fluorophore were injected t.d. into the tibial muscle of *ACVR1*^*R206H/+*^ mice two days (early HO) or twelve days (late HO) after pinch injury. At the early stage of HO formation, DCA-conjugated siRNA accumulated highly in injured muscle areas and infiltrating immune cells (**Extended Fig. 6a**), whereas in the late stage, DCA-conjugated siRNA effectively targeted regenerating muscle fibers and heterotopic bones where osteoblasts and chondrocytes reside (**Extended Fig. 6b**). Next, we determined cell-specific uptake of DCA-conjugated siRNA at early stage of HO using flow cytometry analysis: muscle satellite cells (MuSc, 98.7%), fibroadipogenic progenitors (FAP, 90.8%), B lymphocytes (17.2%), T lymphocytes (16.9%), neutrophils (94.4%), macrophages (95.9%), and dendritic cells (94.7%, Fig. 2b). These results suggest that t.d. administration of DCA-conjugated siRNA at the early stage of HO preferentially accumulates in muscle-resident cells (MuSc, FAP) and innate immune cells with high phagocytic activity (neutrophils, macrophages, dendritic cells), but is less selective toward adaptive immune cells with low phagocytic activity (B and T cells).

To evaluate the ability of DCA-conjugated siACVR1 to suppress HO development in the skeletal muscle of FOP mice, non-targeting control siRNA (siNTC) or siACVR1 were injected t.d. into the tibial muscle of 8-week-old *ACVR1*^*R206H/+*^ mice two days after pinch injury (Fig. 2c). Efficient silencing of *ACVR1*^*R206H*^, without affecting mRNA levels of endogenous mouse *Acvr1*, was confirmed four weeks post-injection (Fig. 2d, **Extended Figure 6c–d**). Compared to the siNTC, siACVR1 significantly reduced HO formation in the injured muscle of *ACVR1*^*R206H/+*^ mice 4 weeks post-treatment (Fig. 2e). These results demonstrate that allele specific silencing of *ACVR1*^*R206H*^ by a single locally delivered siACVR1 effectively suppresses trauma-induced HO in FOP mice. Given our *in vitro* findings that siACVR1 treatment inhibits Activin A-induced chondrogenesis (**Extended Fig. 4e–f**), we examined its ability to suppress chondrogenic anlagen formation during HO pathogenesis by performing histopathological and immunohistochemical (IHC) analyses of siRNA-treated, injured muscle seven days after pinch injury (**Extended Fig. 7**). Compared with siNTC treatment, siACVR1 resulted in a modest reduction in the early chondrogenic gene Sox9, but a significant decrease in the late chondrogenic gene Aggrecan and in cartilage matrix proteoglycans. Collectively, a single t.d. administration of DCA-conjugated siACVR1 into the tibial muscle enables efficient, sustained, and allele specific silencing of *ACVR1*^*R206H*^. This approach inhibits the mutant receptor’s aberrant BMP pathway signaling and subsequent chondrogenic anlagen and heterotopic bone formation in injured muscle of FOP mice.

### Inhibition of IL-1 signaling suppresses trauma-induced HO in FOP

**Figure 3.**
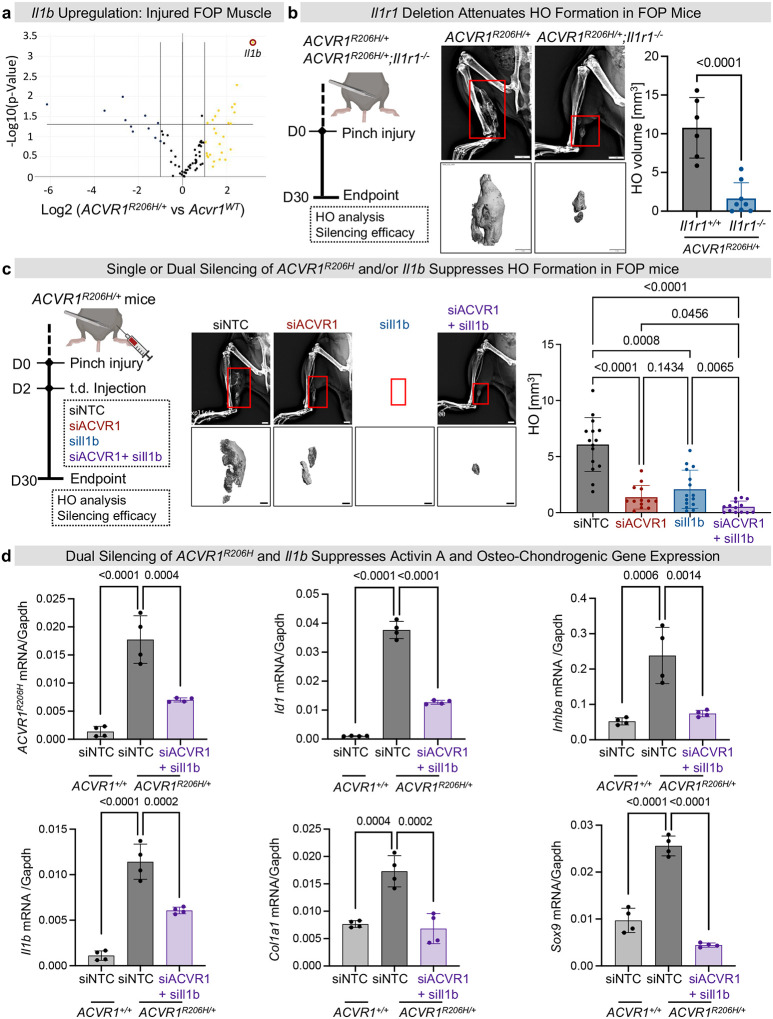
Synergistic effects of combinatory treatment of DCA-conjugated siRNAs targeting *Il1b* and *ACVR1*^*R206H*^ in FOP mice. **a.** 8-week-old *ACVR1*^*+/+*^ and *ACVR1*^*R206H/+*^ mice underwent pinch injury on tibial muscle, and injured tissues were collected at 72 hours post-injury to assess inflammatory gene expression by RT_2_ profiler PCR array (n = 3). A scatter plot was generated from multiple t-test. **b.** 8-week-old *ACVR1*^*R206H/+*^ and *ACVR1*^*R206H/+*^*;Il1r1*^*−/−*^ mice underwent pinch injury on tibial muscle, and 4 weeks later, HO was assessed by microCT imaging and quantification (n = 5–8). **c.** A single dose of siNTC, siACVR1, siIl1b, and siACVR1 + siIl1b (10 nmol) was injected t.d. into the tibial muscle of 8-week-old *ACVR1*^*R206H/+*^ mice 2 days post-injury. 4 weeks later, HO was assessed by microCT imaging and quantification (n = 15). **d.** A single dose of siNTC or siACVR1+siIl1b (10 nmol) was injected t.d. into the tibial muscle of 8-week-old *ACVR1*^*+/+*^*;PDGFRα-GFP* and *ACVR1*^*R206H/+*^*;PDGFRα-GFP* mice 2 days post-injury. 3 days later, GFP^+^Sca1^+^CD31^−^CD45^−^ FAPs were FACS-sorted from digested muscle near the site of injury and subjected to RT-PCR to assess knockdown efficiency of *ACVR1*^*R206H*^ and *Il1b* as well as gene expression (*Id1, Inhba, Col1a1, Sox9*) (n = 4). Scale bars: 5 mm **(b, c)**. Red boxes in the 2D radiographic images indicate 3D microCT images of heterotopic bones within tibial muscle. Data are representative of two independent experiments. Values represent mean ± SD, with statistical significance determined by two-sided unpaired t-tests **(b)** and one-way ANOVA test **(c, d)**.

FOP is characterized by excessive extraskeletal bone formation and recurrent flare-ups, which are associated with elevated levels of inflammatory cytokines and respond to anti-inflammatory treatments^43,44^. To identify inflammatory factors contributing to the initiation of HO, we examined the expression levels of 84 genes involved in inflammatory responses to pinch injury in *ACVR1*^*R206H/+*^ muscle (**Extended Fig. 8a**). More than 25 inflammatory cytokines and chemokines, including *Csf1, Ccl4, Cll7, Ccl12, Cxcl10, Cxcl4*, and *Il1b*, were upregulated in *ACVR1*^*R206H/+*^ muscle compared to *ACVR1*^*+/+*^ muscle, with *Il1b* showing the most prominent induction (Fig. 3a, **Extended Fig. 8a**). These genes are involved in the recruitment of innate immune cells, mesenchymal stem cells, and progenitors to injury sites, thereby initiating HO pathogenesis. To assess the kinetics of *Il1b* expression in injured muscle tissue, *ACVR1*^*+/+*^ and *ACVR1*^*R206H/+*^ mice were subjected to pinch injury, and muscle tissue was harvested at 3, 6, 24, and 72 hours post-injury. *Il1b* expression peaked at 6 hours after injury and gradually decreased over time; however, compared to *ACVR1*^*+/+*^ muscle, *ACVR1*^*R206H/+*^ muscle demonstrates significantly increased expression throughout the time course (**Extended Fig. 8b**). This is consistent with FOP patients showing heightened sensitivity to injury and elevated *Il1b* levels during flare-ups^20^. Notably, an increased pro-inflammatory state characterized by enhanced NF-κB and p38 MAPK activity has been observed in primary monocytes isolated from FOP patients^45^. *ACVR1*^*R206H*^ BMSCs also showed a significant increase in *Il1b* expression in response to pro-inflammatory stimuli such as TNF, IL-1β, and the Toll-like receptor 4 activator lipopolysaccharide (LPS) (**Extended Figure 8c**). Collectively, these results suggest that hyperactivation of NF-κB and p38 MAPK signaling downstream of TNF, IL-1β, and LPS may promote further expression of *Il1b* in *ACVR1*^*R206H*^ BMSCs, potentially fueling the auxiliary inflammatory cascade that contributes to HO initiation.

Given that IL-1 blockers significantly reduce the frequency of flare-ups and HO in FOP patients^23^, we investigated whether inhibiting IL-1 signaling through genetic deletion of the IL-1 receptor 1 (*Il1r1*) could dampen inflammation and HO in FOP mice (Fig. 3b). We assessed inflammatory gene expression at an early stage of HO (3 days post-injury, **Extended Fig. 8d**) and heterotopic bone formation at a later stage (4 weeks post-injury, Fig. 3b) in injured muscle of *ACVR1*^*R206H/+*^ mice crossed with *Il1r1*^−/−^ mice (*ACVR1*^*R206H/+*^*; Il1r1*^*−/−*^). Expression of multiple cytokines and chemokines (**Extended Fig. 8d**) and *Inhibin A* (*Inhba*)—a protein subunit of *Activin A* homodimer (**Extended Fig. 8e**)—were dampened in the absence of IL1R1, suggesting that IL-1 signaling may play a role in regulating expression of various cytokines/chemokines and Activin A during HO progression.

Next, we designed and synthesized a panel of candidate siRNAs targeting *Il1b* to inhibit IL-1 signaling. Since *Il1b* expression is upregulated in mouse monocytes/macrophages upon LPS stimulation^46^, an initial primary screening was conducted in LPS-treated mouse Raw 264.7 monocyte cells. The sequences and chemical modification patterns are detailed in **Extended Table 3**. Compared to siNTC, six siRNA sequences (872, 1117, 1128, 1245, 1293, 1296) showed 80–85% silencing efficiency of *Il1b* mRNA (**Extended Fig. 9a**). Among them, siRNA_1293, herein referred to as siIl1b, was identified as the most potent compound after further evaluation in both Raw 264.7 cells (**Extended Fig. 9b**) and primary bone marrow-derived macrophages (BMDM, **Extended Fig. 9c**) in a 7-point dose-response assay.

Following the identification of a lead compound targeting mouse *Il1b*, a single dose of siACVR1, siIl1b, or their combination was administered t.d. into the tibial muscle of *ACVR1*^*R206H/+*^ mice two days post-injury (Fig. 3c). Four weeks later, the knockdown efficiency of *ACVR1*^*R206H*^ and *Il1b* in injured muscle was confirmed (**Extended Fig. 10a**). Similar to the effects observed with genetic deletion of IL-1 receptor, siRNA-mediated silencing of *Il1b* significantly reduced HO formation, comparable to the suppression achieved by allele-specific silencing of *ACVR1*^*R206H*^ (Fig. 3c). Remarkably, combined treatment with siACVR1 and siIl1b nearly abolish HO formation (Fig. 3c), indicating a cooperative effect of siRNA-mediated inhibition of both ACVR1^R206H^ receptor and IL-1 signaling pathways in FOP. These findings are also supported by histopathological and IHC analyses of siRNA-treated, injured muscle seven days after pinch injury. Compared with siNTC treatment, siIl1b and the combination of siACVR1 and siIl1b markedly reduced the expression of chondrogenic genes and the production of cartilage matrix proteoglycans (**Extended Fig. 7**). Together, these results demonstrate that treating siIl1b alone or in combination with siACVR1 effectively suppress chondrogenic anlagen and heterotopic bone formation in injured muscle of FOP mice.

To further assess the capacity of these siRNAs to suppress FAP-driven HO, Pdgfrα^+^ FAPs were isolated from the injured muscle of *ACVR1*^*+/+*^*;Pdgfrα-GFP* and *ACVR1*^*R206H/+*^*;Pdgfrα-GFP* mice three days after siRNA injection using GFP expression. Compared to siNTC-treated WT FAPs, siNTC-treated FOP FAPs showed significant upregulation of *Il1b* (inflammatory gene), *Inhba* (Activin A gene), *Id1* (BMP-responsive gene), and *Col1α1* (osteogenic gene). Notably, this induction was markedly reduced by treatments with siACVR1, siIl1b, or their combination (Fig. 3d, **Extended Fig. 10b**). These findings suggest that both single and dual silencing of *ACVR1*^*R206H*^ and *Il1b* effectively suppresses aberrant BMP signaling and inflammation, thereby reducing osteogenesis in muscle resident FAPs during HO progression. Thus, inhibiting ACVR1^R206H^ and IL-1 signaling via combined administration of siACVR1 and siIl1b presents a promising strategy to more effectively prevent the initiation of traumatic HO in FOP.

### Development of a novel unimolecular, DCA-conjugated, divalent siRNA for dual silencing of ACVR1^R206H^ and Il1b

**Figure 4.**
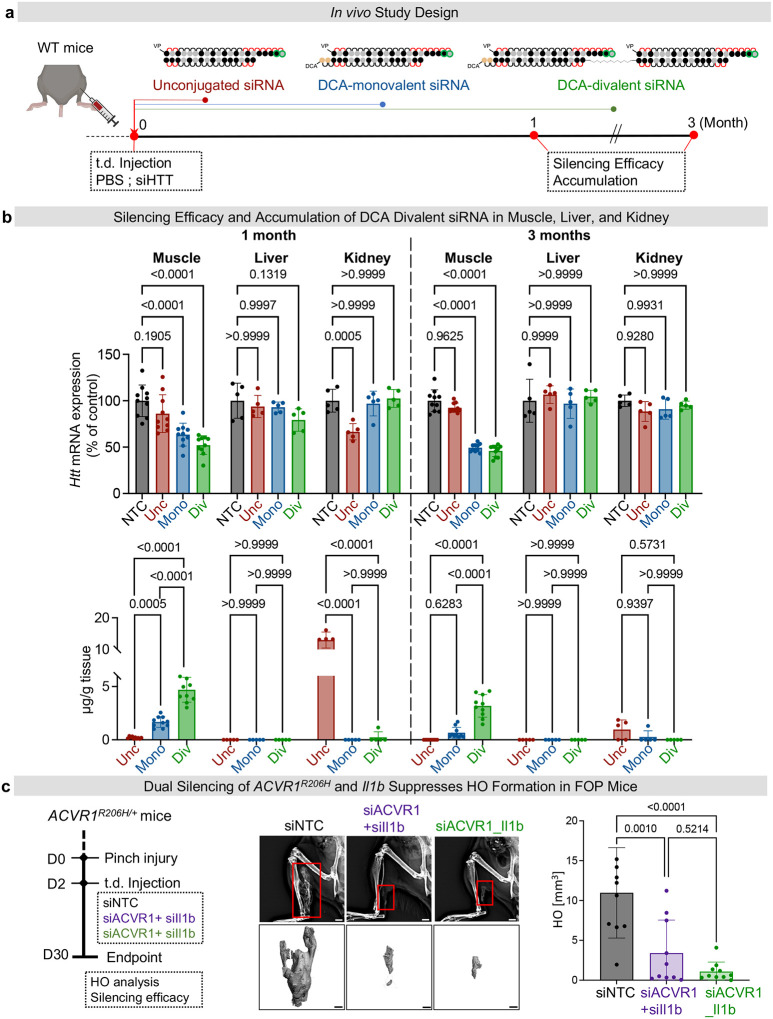
Development of a unimolecular DCA-conjugated divalent siRNA targeting *ACVR1*^*R206H*^ and *Il1b*. **a, b.** Schematic diagram showing study design (**a**). A single dose of Cy3-labeled unconjugated monovalent (Unc) siRNA, DCA-conjugated monovalent (DCA Mono) siRNA, or DCA-conjugated divalent (DCA Div) siRNA (10 nmol) was injected t.d. into the tibial muscle of 8-week-old wildtype mice. 1- and 3-months later, knockdown efficiency (**b, top**) and antisense accumulation (**b, bottom**) were assessed (n = 5). **c.** A single dose of siNTC, siACVR1+Il1b, or siACVR1_Il1b (10 nmol) was injected t.d. into the tibial muscle of 8-week-old *ACVR1*^*R206H/+*^ mice 2 days post-injury. 4 weeks later, HO was assessed by microCT imaging and quantification (n = 10). Red boxes in the 2D radiographic images indicate 3D microCT images of heterotopic bones within tibial muscle. Scale bars: 5 mm. Data are representative of two independent experiments. Values represent mean ± SD, with statistical significance determined by one-way ANOVA test **(b, c)**.

The design of a combinatorial, monovalent siRNA therapeutic approach targeting *ACVR1*^*R206H*^ and *Il1b* has proven effective in suppressing HO formation in FOP mice. However, given the high activity of both ACVR1^R206H^ and IL-1 signaling in both FAP and immune cells, we hypothesized that developing a unimolecular structure capable of simultaneously targeting both pathways could further enhance therapeutic efficacy. Additionally, synthesis of a single molecule would greatly simplify pharmaceutical translation in the future. To this end, we have developed a first-in-class, DCA-conjugated divalent siRNA scaffold by linking two siRNAs together via a tetraethylene glycol (TEG)^47^. This unimolecular dual-targeting siRNA enables the concurrent delivery of both siRNAs at equimolar amounts directly to skeletal muscle, FAPs, and immune cells while also simplifying efficacy, durability, off-target assessment, and safety evaluations (Fig. 4a).

To compare tissue biodistribution, durability, and knockdown efficiency of the DCA-conjugated divalent siRNA against traditional DCA-conjugated monovalent siRNA, Cy3-labeled DCA-conjugated monovalent and divalent siRNAs targeting the ubiquitously expressed *Huntington* (*Htt)* gene were injected t.d. into the tibial muscle. One or three months later, tissue biodistribution and durability were visualized using an IVIS optical imaging system (**Extended Fig. 11a**). The knockdown efficiency was also confirmed via Quantigene 2.0 assay (Fig. 4b, **top**), while antisense strand accumulation was quantified by PNA hybridization assay (Fig. 4b, **bottom**). Our results showed that three months after a single administration, Cy3 fluorescence, antisense strand accumulation, and *Htt* mRNA silencing were predominantly retained in the tibial muscle with little to no leakage into the liver and kidney. Importantly, the DCA-conjugated divalent siRNA exhibited significantly higher muscle retention, Cy3 signal intensity, and antisense accumulation than its monovalent counterpart, demonstrating the superior stability and sustained delivery afforded exclusively by this novel divalent structure. Next, we examined the cell types targeted by DCA-conjugated divalent siRNA in the injured muscle of FOP mice. DCA-conjugated divalent siRNA was injected t.d. into the tibial muscle of *ACVR1*^*R206H/+*^ mice two days (early HO stage) or twelve days (late HO stage) post-injury. Similar to DCA-conjugated monovalent siRNA, DCA-conjugated divalent siRNA demonstrated high accumulation in injured muscle and infiltrating immune cells at the early stage of HO. FACS analysis revealed substantial siRNA uptake in various cell types, including MuScs (96.5%), FAPs (84.6%), B lymphocytes (12.9%), T lymphocytes (13.8%), neutrophils (88.6%), macrophages (90.8%), and dendritic cells (95.6%, **Extended Fig. 11b-c**). At the later stage of HO, DCA-conjugated divalent siRNA also effectively targeted osteoblasts and chondrocytes within the heterotopic bone (**Extended Fig. 11d**). These findings demonstrate that local delivery of DCA-conjugated divalent siRNA efficiently transfects muscle-resident cells (MuSc, FAP), innate immune cells (neutrophils, macrophages, dendritic cells), and HO-forming cells (chondrocytes and osteoblasts) throughout HO development.

To compare the therapeutic efficacy of the unimolecular DCA-conjugated divalent siRNA targeting *ACVR1*^*R206H*^ and the *Il1b* (siACVR1_Il1b) vs the combination of DCA-conjugated monovalent siRNAs targeting *ACVR1*^*R206H*^ and the *Il1b* (siACVR1 + siIl1b) in FOP, siRNAs were injected t.d. into the tibial muscle of *ACVR1*^*R206H/+*^ mice three days post-injury (Fig. 4c). Four and eight weeks later, the knockdown efficiency of *ACVR1*^*R206H*^ and *Il1b* mRNA in the muscle was validated (**Extended Fig. 12a and 13a**). Both siACVR1 + siIl1b and siACVR1_Il1b treatments demonstrated effective suppression of HO formation in injured muscle (Fig. 4c) and this inhibitory effect was maintained at eight weeks post-treatment (**Extended Figure 13b and c**), suggesting that each approach comparably inhibits trauma-induced HO. To examine siRNA’s effects in FOP at cellular levels, Pdgfrα^+^ FAPs and lineage-positive immune cells—including T and B lymphocytes, NK cells, monocytes, macrophages, and granulocytes—were isolated from injured muscle of *ACVR1*^*+/+*^*;PDGFRα-GFP* (WT) and *ACVR1*^*R206H/+*^*;PDGFRα-GFP* (FOP) mice. Compared to siNTC-treated WT-FAPs, siNTC-treated FOP-FAPs exhibited a significant increase in expression of *ACVR1*^*R206H*^*, Il1b*, and *Inhba*. This elevated expression was reduced by treatment with either siACVR1 + siIl1b or siACVR1_Il1b (**Extended Fig. 12b**). Similarly, the heightened *ACVR1*^*R206H*^*, Il1b,* and *Inhba* expression in immune cells was also substantially decreased following treatment with siACVR1 + siIl1b or siACVR1_Il1b (**Extended Fig. 12c**). These results indicate that both FAPs and immune cells in FOP muscle upregulate expression of *Il1b*, *Activin A,* and *ACVR1*^*R206H*^ in response to injury (danger signal), and that dual silencing of *ACVR1*^*R206H*^ and *Il1b* effectively suppresses IL-1-mediated inflammation, aberrant BMP signaling pathways, and HO formation.

### Reversal of gene expression profiles in FOP FAPs by dual silencing of ACVR1^R206H^ and Il1b

**Figure 5.**
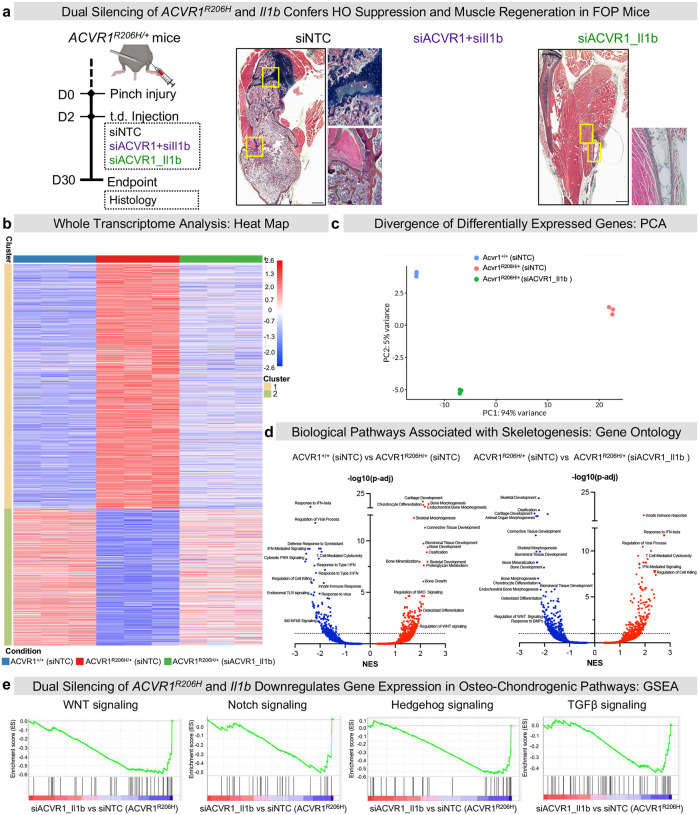
Molecular impacts of DCA-conjugated divalent siRNA targeting *ACVR1*^*R206H*^ and *Il1b* in FAP-driven HO. **a.** A single dose of siNTC, siACVR1+Il1b, or siACVR1_Il1b (10 nmol) was injected t.d. into the tibial muscle of 8-week-old *ACVR1*^*R206H/+*^ mice 2 days post-injury. 4 weeks later, HO lesions were stained with alcian blue (n = 10). Scale bars: 200 μm. **b-e.** A single dose of siNTC or siACVR1_Il1b (10 nmol) was injected t.d. into the tibial muscle of *ACVR1*^*+/+*^*;PDGFRα-GFP* and *ACVR1*^*R206H/+*^*;PDGFRα-GFP* mice 2 days post-injury. 5 days later, GFP^+^Sca1^+^CD31^−^CD45^−^ FAPs were FACS-sorted from digested muscle near the site of injury and then, subjected to RNA sequencing (n = 3). Heatmap displaying gene expression patterns with k-means clustering (k=2) of differentially expressed genes (adjusted p-value ≤ 0.05, |log2FoldChange| ≥ 1) across the three treatment groups. Expression values are centered and variance-stabilized. Genes are grouped by cluster membership, with samples ordered by treatment condition **(b)**. Principal component analysis (PCA) plot illustrating sample clustering based on variance-stabilized gene expression data (**c**). Volcano plots show biological pathways with significantly upregulated and downregulated gene expression for pairwise comparisons. Gene ontology (GO) analysis reveals multiple biological processes associated with bone formation and immune responses (**d**). Gene set enrichment analysis (GSEA) shows enrichment of osteo-chondrogenic genes involved in WNT, Notch, Hedgehog, and TGFβ signaling **(e)**. Data are representative of two independent experiments.

To further understand the cellular and molecular mechanisms underlying the effects of dual silencing siRNAs of *ACVR1*^*R206H*^ and *Il1b* in FOP, histologic analysis was performed on injured muscle of FOP mice four weeks post-injury (Fig. 5a, **left**). Unlike siNTC-treated *ACVR1*^*R206H/+*^ muscles—which exhibited established heterotopic bone, cartilage matrix proteoglycans, and a bone marrow compartment—siACVR1 + siIl1b—or siACVR1_Il1b—treated muscles were almost completely regenerated from injury with little to no chondrogenic analgen and heterotopic bone formation (Fig. 5a, **right**). To ensure that the natural trauma-induced muscle degeneration process required for wound healing remains intact, histological analysis was performed four days after injury, revealing normal muscle degeneration in FOP mice following treatment with siACVR1 + siIl1b or siACVR1_Il1b (**Extended Fig. 14a-b**). Next, flow cytometry analysis was conducted seven days post-injury to examine muscle and immune cell populations in the siRNA-treated, injured muscle of FOP mice. These results demonstrate that treatment with siACVR1 + siIl1b or siACVR1_Il1b reduced the frequencies of muscle-resident cells, FAPs and MuScs, as well as neutrophils, and B lymphocytes. However, monocyte populations were significantly increased, whereas inflammatory macrophages and T lymphocytes remained unchanged (**Extended Fig. 14c**). These results indicate that dual silencing of *ACVR1*^*R206H*^ and *Il1b* in injured muscle reshapes the cellular composition of FAPs, MuScs, and immune cell infiltrates without affecting trauma-induced muscle degeneration, thereby contributing to the preservation of muscle regeneration and suppressing HO formation in FOP mice.

To gain insights on molecular mechanisms of siACVR1_Il1b treatment in FAP-driven HO in FOP mice, Pdgfrα^+^ FAPs were FACS-sorted from the injured muscle of WT (*ACVR1*^*+/+*^) and FOP (*ACVR1*^*R206H/+*^) mice five days after siACVR1_Il1b treatment and then, subjected to RNA sequencing (**Extended Fig. 15a**). We performed a likelihood ratio test to analyze interaction effects between: (1) siNTC-treated WT vs FOP FAPs, (2) siNTC- vs siACVR1_Il1b-treated FOP FAPs, and (3) siNTC-treated WT FAPs vs siACVR1_Il1b-treated FOP FAPs. Subsequent k-means clustering revealed that genes were significantly affected (FDR ≤ 0.05), forming two clusters: cluster one was associated with genes that were upregulated by the R206H mutation, and were effectively blunted by siACVR1_Il1b in FOP FAPs, while cluster two was associated with genes downregulated by the R206H mutation, their suppression minimized upon siACVR1_Il1b treatment (Fig. 5b).

Principal component analysis (PCA) showed that siNTC**-**treated FOP FAPs were transcriptomically distinct from siNTC-treated WT FAPs; however, this divergence was reduced by siACVR1_Il1b treatment in FOP FAPs (Fig. 5c). Gene ontology analysis indicated that genes within cluster one are enriched in morphogenetic and skeletogenic processes, especially bone, cartilage, and connective tissue development. Notably, genes involved in chondrogenesis, osteogenesis, inflammation, and wound healing were highly upregulated in FOP FAPs compared to WT FAPs but were significantly downregulated following siACVR1_Il1b treatment (Fig. 5d). Upregulation of BMP-responsive genes, including *Bambi, Id1, Id3*, and *Noggin,* in siNTC-treated FOP FAPs was blunted by siACVR1_Il1b treatment (**Extended Fig. 15b**). Additionally, gene set enrichment analysis (GSEA) revealed suppression of key signaling pathways—WNT, Notch, Hedgehog, and TGFβ**—**that drive osteo-chondrogenic differentiation of FAPs (Fig. 5e). These findings demonstrate that siACVR1_Il1b effectively inhibits early transcriptional programs associated with chondrogenesis, osteogenesis, and inflammation in FOP FAPs, supporting its potential as a therapeutic strategy to block inflammation-driven differentiation of FAPs and HO formation in FOP.

### Systemic delivery of siRNAs targeting ACVR1^R206H^ and Il1b suppresses HO in FOP

While local siRNA delivery effectively reduces trauma-induced HO in FOP mice, systemic administration enables targeting of multiple lesion sites throughout the body. To assess biodistribution of DCA-conjugated monovalent and divalent siRNAs in individual tissues, DCA-conjugated, monovalent or divalent siRNAs were administered subcutaneously (s.c.) to *ACVR1*^*R206H/+*^ mice one day post injury (**Extended Fig. 16a**). One week later, siRNA accumulation (**Extended Fig. 16b**) and silencing efficiency (**Extended Fig. 16c**) were evaluated. These results demonstrate that systemic delivery of both siRNAs showed broad distribution across multiple tissues in FOP mice, with robust silencing efficiency in muscle and heart and present in clearance organs such as the liver. Next, we determined cell-specific uptake of DCA-conjugated siRNAs in injured muscle using flow cytometry analysis (**Extended Fig. 16d-e**). FACS analysis revealed substantial uptake of both monovalent and divalent siRNAs in MuSCs (98% monovalent, 98% divalent), FAPs (98% monovalent, 99% divalent), and inflammatory myeloid cells (97% monovalent, 92% divalent). These results suggest that, similar to t.d. administration, s.c. administration of DCA-conjugated monovalent and divalent siRNAs at the early stage of HO preferentially accumulates in muscle-resident cells (MuSc, FAP) and inflammatory myeloid cells.

**Figure 6.**
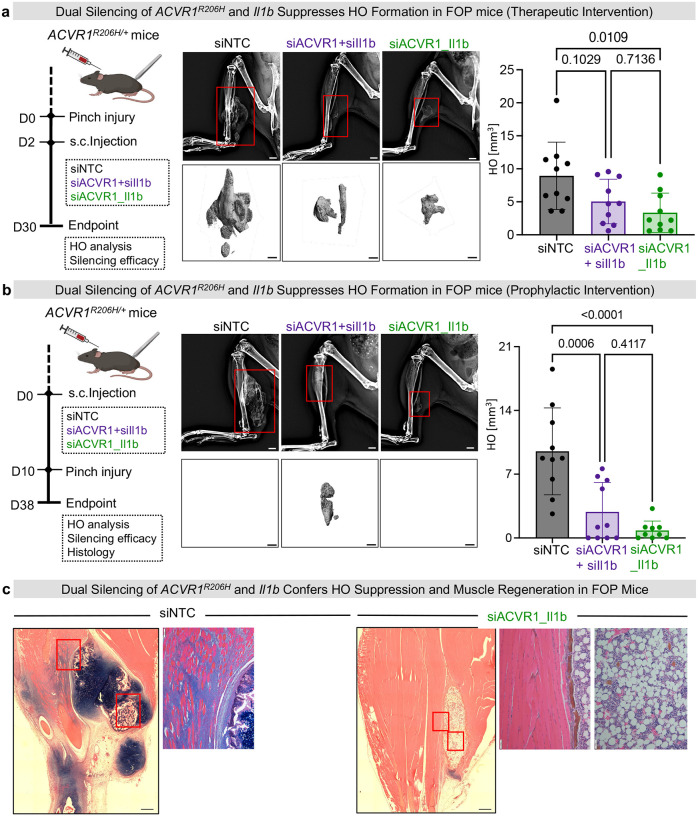
Systemic delivery of DCA-conjugated siRNAs targeting *ACVR1*^*R206H*^ and *Il1b* suppresses HO in FOP mice. **a.** A single dose of siNTC, siACVR1+siIl1b, or siACVR1_Il1b (40 mg/kg) was injected subcutaneously (s.c.) into 8-week-old *ACVR1*^*R206H/+*^ mice 1 day post-injury. 4 weeks later, HO was assessed by microCT imaging and quantification (n = 10). **b, c.** A single dose of siNTC, siACVR1+siIl1b, or siACVR1_Il1b (40 mg/kg) was injected s.c. into 8-week-old *ACVR1*^*R206H/+*^ mice 10 days prior to pinch injury. 4 weeks later, HO was assessed by microCT imaging and quantification (n = 10, **b**). Alternatively, HO lesions were stained with alcian blue (**c**), demonstrating the ability of siACVR1_Il1b to suppress HO. Red boxes in the 2D radiographic images indicate 3D microCT images of heterotopic bones within tibial muscle. Scale bars: 5 mm **(a, b)**, 200 μm **(c)**. Data are representative of two independent experiments. Values represent mean ± SD, with statistical significance determined by one-way ANOVA test **(a, b)**.

To evaluate whether systemic delivery of dual silencing siRNAs of *ACVR1*^*R206H*^ and *Il1b* can suppress HO in FOP mice, a single dose of siACVR1 + siIl1b or siACVR1_Il1b was administered via s.c. injection into *ACVR1*^*R206H/+*^ mice two days post-injury (Fig. 6a). Four weeks later, knockdown efficiency of *ACVR1*^*R206H*^ and *Il1b* in various tissues was examined (**Extended Fig. 17a**). siACVR1_Il1b treatment significantly reduced HO formation, whereas only a modest decrease was observed in siACVR1 + siIl1b-treated muscle; however, the two were not statistically significant from one another (Fig. 6a). This attenuated effect is likely attributable to both limited accumulation in the target muscle following systemic administration, compared with levels achieved by local injection, and/or a potential delay in onset of gene silencing after systemic delivery.

Next, we tested whether systemically administered prophylactic siRNA treatment could attenuate HO formation in FOP miceA single dose of siACVR1 + siIl1b or siACVR1_Il1b was injected s.c. into *ACVR1*^*R206H/+*^ mice ten days prior to injury (Fig. 6b). Histological analysis was performed four days after injury, demonstrating that trauma-induced muscle degeneration process required for wound healing remains intact in siRNA-treated mice **(Extended Fig. 17b).** Four weeks post-injury, efficient knockdown efficiency of *ACVR1*^*R206H*^ and *Il1b* in muscle and liver was examined (**Extended Fig. 17c**). Both treatment groups showed a significant reduction in HO formation at the injury site (Fig. 6b). This is consistent with histological analysis of FOP mice showing that siNTC-treated muscle developed heterotopic bone with a marrow cavity via endochondral ossification, along with fibrotic tissue and chondrogenic anlagen within neighboring muscle. In contrast, siACVR1_Il1b-treated muscle exhibited a significant reduction in heterotopic bone size, which was filled with adipose tissue internally and surrounded by intact, regenerated muscle tissue (Fig. 6c). Of note, systemic treatment with either siACVR1 + siIl1b or siACVR1_Il1b did not affect circulating lymphocytes and monocytes frequencies or red blood cell and platelet counts (**Extended Fig. 18a**). Additionally, histological analysis demonstrated normal tissue morphology and structure of the heart, lung, muscle, liver, spleen, and kidney in these mice (**Extended Fig. 18b**). Collectively, these results demonstrate that prophylactic systemic administration of combined siACVR1 and siIL1b siRNAs or siACVR1_IL1b achieves effective dual silencing of *ACVR1*^*R206H*^ and *Il1b* in skeletal muscle and suppresses HO pathogenesis in FOP mice.

To further assess safety, siACVR1, siIl1b, or siACVR1_Il1b was administered s.c. to wild-type mice and seven days later, complete blood counts and blood chemistry were performed (**Extended Fig. 19**). None of the siRNAs altered circulating white blood cell populations, including lymphocytes, monocytes, and neutrophils, nor did the siRNAs affect red blood cells and platelet and hemoglobin levels relative to PBS controls. No evidence of anemia was observed, and hepatic toxicity biomarkers, including alkaline phosphatase [ALP] and alanine aminotransferase [ALT], remained unchanged. In addition, renal function biomarkers, including blood urea nitrogen [BUN], calcium, and phosphate, showed little to no differences between groups. Thus, prophylactic systemic administration of siACVR1, siIl1b, or siACVR1_Il1b suppresses HO formation in FOP mice, is well tolerated, and does not cause detectable systemic toxicity or adverse effects in non-target tissues in mice. This approach provides a promising, effective, and safe strategy for suppressing debilitating HO, providing a compelling proof-of-concept for clinical translation to FOP patients.

### Validation of human IL1B silencing and ACVR1 allele selectivity in human muscle tissues

We next assessed the efficacy and selectivity of siRNAs in a human context. Due to limited sequence homology between mouse *Il1b* and human *IL1B*, the mouse targeting siIl1b sequence does not knockdown human *IL1B* expression. We therefore designed and synthesized a panel of candidate siRNAs targeting human *IL1B*. The sequences and chemical modification patterns are detailed in **Extended Table 4.** Primary screening in LPS-stimulated human lymphoblastic cell line HEL92.1.7. identified ten siRNA sequences (51, 140, 1399, 1344, 1356, 1362, 1387, 1418, 1425, 1453) showing greater than 60% silencing efficiency of human *IL1B* mRNA (**Extended Fig. 20a**). Among them, siRNA_1339, herein referred to as siIL1B, was identified as the most potent compound after further evaluation in both HEL92.1.7. cells (**Extended Fig. 20b**) and primary peripheral blood mononuclear cells (PBMCs, **Extended Fig. 20c**) in a 7-point dose-response assay. Finally, we obtained noncancerous excess human quadriceps muscle tissue discarded during a planned, unrelated sarcoma tumor excision surgery. Tissues were cultured *ex vivo* and treated with PBS or siRNAs at various concentrations. Four days later, silencing efficacy was assessed by the QuantiGene 2.0 assay (**Extended Fig. 20d, right**), demonstrating dose dependent response with potent silencing of human *IL1B* reaching greater than 80% at the highest dose.

ACVR1-mediated signaling plays a critical role in a broad range of biological processes, including bone development and homeostasis.^16^ We therefore examined whether siACVR1 treatment preserves wild-type ACVR1 expression in fresh human skeletal muscle tissues, and observed limited silencing of the wild-type human *ACVR1* allele with significant silencing only occurring at the highest dose with ~43% reduction of wildtype ACVR1 mRNA (**Extended Fig. 20d, left**). Together with our data from FOP patient-derived iPSCs (Fig. 1d), these results demonstrate that siACVR1 selectively silences the mutant *ACVR1*^*R206H*^ allele expression while preserving wild-type *ACVR1* expression in human iPSCs and muscle tissues. Combined with the extensive efficacy testing performed in an FOP mouse model, this validation in human tissue supports the potential of siACVR1_IL1B as a novel therapeutic for clinical translation. Taken together, siRNA therapy provides a promising, effective, and safe strategy for suppressing debilitating HO, providing a compelling proof-of-concept for clinical translation to FOP patients.

## Discussion

FOP is a devastating ultra-rare disorder caused by a single nucleotide mutation that drives spontaneous or trauma-induced HO. Current investigational therapies for FOP include Ipsen’s FALKON trial with fidrisertib (an ALK2/ACVR1 inhibitor), Regeneron’s OPTIMA program with garetosumab (an Activin A monoclonal antibody), STOPFOP with saracatinib (a Src family kinase inhibitor), and Ipsen’s repurposing trial with palovarotene (a retinoid γ agonist). However, these approaches neither target nor correct the underlying ACVR1 mutation, and their long-term use may be limited by adverse effects. In contrast, siRNA therapy selectively silences mutant *ACVR1*^*R206H*^ expression while largely sparing wild-type *ACVR1* expression, thereby minimizing off-target toxicity. In addition, IL-1β silencing attenuates the inflammatory triggers of flare-ups, providing a dual-mechanism approach that combines durable inhibition of aberrant BMP signaling and inflammation. Unlike one-time adeno-associated virus (AAV)-based gene therapies, which can be constrained by immune responses, siRNA therapeutics also enable repeat systemic dosing at defined intervals with minimal adverse events.

Here, we describe the development and validation of a novel unimolecular divalent siRNA targeting mutant ACVR1^R206H^ and IL-1β for FOP treatment. We demonstrate that suppressing both inflammation and the causative ACVR1^R206H^ mutation ablate HO pathogenesis, with combined targeting providing the greatest therapeutic benefit. The molecule developed herein provides disease-modifying effects after both local and systemic administration in preventive and therapeutic models, supporting its potential use in the treatment of FOP and the broader potential of this platform for musculoskeletal disorders requiring simultaneous modulation of multiple genes or pathways.

### SNP-selectivity

ACVR1 is an essential gene involved in a broad range of biological processes, including bone development and homeostasis^16^, making allele-selective therapeutic targeting necessary. Population-level data from gnomAD suggest that loss of function in a single *ACVR1* allele is generally well tolerated^48^ and mice with heterozygous deletion of *Acvr1* do not exhibit any gross abnormalities^49^, therefore *ACVR1*^*R206H*^ allele-specific silencing likely represents a safe and promising therapeutic approach for FOP. In this study, we achieved selective targeting of *ACVR1*^*R206H*^ allele using *in vitro* reporter assays and FOP patient-derived iPSCs that express heterozygous *ACVR1*^*R206H*^ allele. Because obtaining large amounts of muscle tissues from FOP patients poses a substantial risk of inducing additional HO, we utilized patient iPSCs to determine SNP selectivity instead of ideal assessment using patient muscle tissues. While *ex vivo* analyses in healthy human muscle tissues provided initial evidence regarding lack of non-specific silencing, these systems do not adequately model systemic pharmacology, long-term toxicity, or therapeutic efficacy *in vivo*. Therefore, future clinical studies will also need to incorporate longitudinal monitoring of wild-type ACVR1 expression to further assess the impact of any allelic off targeting in patients receiving treatment.

### Inflammation in FOP

Our data indicate that co-modulating inflammation and aberrant ACVR1^R206H^ signaling yields an improved therapeutic response. While previous studies have implicated inflammation as a trigger of aberrant ACVR1^R206H^ signaling^45^, with IL-1 signaling suggested as a potential therapeutic target^20,23^, our findings are the first to suggest that IL-1-mediated signaling contributes to trauma-induced HO development in FOP. The observed benefit of combined targeting supports its role as a direct modulator of disease severity.

Three IL-1 blockers are currently approved for treating patients with chronic inflammatory diseases, including rheumatoid arthritis and autoinflammatory diseases. In addition, these agents have been used in pediatric FOP patients as part of ongoing clinical management. Across these patient populations, they have demonstrated a favorable safety profile, with generally mild adverse effects. Anakinra is a recombinant human IL-1 receptor antagonist that inhibits both IL-1α and IL-1β binding to the IL-1 receptor and is delivered by daily s.c. injections^50^. Rilonacept is a dimeric Fc fragment containing the extracellular residues of IL-1R1 and IL-1RAcP that inhibits both IL-1α and IL-1β binding to the IL-1 receptor and is delivered by weekly s.c. injections^51^. Canakinumab is a fully humanized anti-IL-1β monoclonal antibody that selectively inhibits IL-1β and is delivered by monthly s.c. injections^52^. Unlike these IL-1 blockers, other immune suppressors, such as JAK inhibitors (e.g., tofacitinib, baricitinib) and TNF inhibitors (e.g., adalimumab, infliximab, etanercept), are associated with more severe adverse effects and require closer monitoring^53,54^. Thus, IL-1-targeted therapies appear to have a more favorable safety profile than other anti-inflammatory agents, suggesting that siRNA-based therapy targeting IL-1β may likewise be well tolerated.

Consistent with this favorable safety profile, our *in vivo* studies similarly showed no detectable effects on hematologic parameters or markers of renal and hepatic toxicity. All this being said, additional investigation may be needed to better define the long-term safety profile of coordinated inflammatory modulation, particularly in pediatric patients with FOP who may require chronic or lifelong treatment. Although suppression of inflammatory signaling may provide therapeutic benefit during flare-associated lesion formation, the consequences of sustained immune modulation over extended treatment periods remain incompletely understood. Longitudinal preclinical studies will therefore be necessary to establish the therapeutic window, define safety margins, and determine the extent to which chronic anti-inflammatory intervention is required for disease control. These data may be collected from ongoing clinical application with above mentioned IL-1 targeting therapeutics, providing an important preliminary insight into the safety of sustained immune modulation, and the extent to which inflammatory modulation in FOP is needed.

### A novel muscle targeting unimolecular, multivalent siRNA chemically architecture.

The use of longer fatty acid tails, such as DCA, enhances serum protein binding and enables robust delivery and functional gene silencing in multiple tissues and cell types, including muscle^34^. Here we show that lipophilic conjugates like DCA can efficiently deliver siRNA cargo of varying sizes. Moreover, divalent lipophilic siRNAs showed enhanced accumulation following local administration and a trend toward increased efficacy with systemic dosing, making this phenomenon worthy of further investigation. We previously demonstrated that increasing valency by itself significantly enhances delivery and tissue distribution following local administration in the CNS^30^, eye^31^, and lung^55^. While both multivalency^30^ and lipophilic conjugation^32^ have been individually studied as PK-modifying strategies, this report is the first description of the combined effects of lipophilicity and size on oligonucleotide PK/PD.

Changing the lipid-to-phosphodiester charge ratio does not affect systemic tissue distribution, which is largely driven by serum binding^56^, and may even enhance endosomal escape^57^, potentially overcoming a key barrier to siRNA efficacy. The use of a unimolecular dual-targeting scaffold offers additional advantages, including simplified manufacturing and development, as well as the biological benefit of ensuring both therapeutic sequences reach the same cell. This may be critical when co-regulation of targets is required at the single-cell level. In FOP, inhibition of both IL-1 and ACVR1^R206H^ signaling pathways in FAPs as well as immune cells is likely beneficial, because IL-1β drives inflammation^58,59^ and Activin A activates aberrant BMP signaling via ACVR1^R206H^ receptor^60^. This may explain the observed trend toward greater functional efficacy compared to co-administration.

Recent advances in siRNA targeting to muscle use an alternative delivery approach, transferrin receptor 1 (TfR1)-targeted antibody–siRNA conjugates^61^. While this strategy offers enhanced muscle selectivity, such specificity may be a drawback for FOP treatment. Effective modulation of target gene expression, particularly of inflammatory pathways, likely requires engagement of multiple cell types, including muscle-resident FAPs, ligament, tendon, and infiltrated immune cells. Therefore, the broader biodistribution of DCA-conjugated siRNAs may offer a therapeutic advantage in this context.

### Translational and Clinical Considerations

siRNA therapeutics offer several intrinsic advantages that make them well suited for the treatment of chronic and dynamic diseases such as FOP. RNA interference enables transient, sequence-specific gene silencing without permanent genomic modification, thereby minimizing the risk of irreversible off-target effects associated with gene editing approaches. This pharmacologic profile allows for repeat dosing, dose titration, and, if necessary, treatment discontinuation to precisely control the extent and duration of target suppression. Consistent with this, prior work from our group using the same DCA-conjugated platform demonstrated no observable safety concerns following repeated administration of 40 mg/kg every two weeks for six months^62^, supporting the tolerability of sustained dosing. Such flexibility may be particularly advantageous in FOP, where disease activity is episodic and may require adaptable intervention across both preventive and reactive settings. Moreover, the durability of siRNA-mediated silencing, often persisting for weeks to months depending on tissue context and delivery strategy, enables sustained target engagement with relatively infrequent dosing. These features position siRNA as a controllable and reversible therapeutic modality with the potential to balance long-term efficacy and safety in the treatment of FOP.

All clinically approved siRNAs are delivered to the liver and primarily rely on multivalent GalNAc conjugation^55^. Lipophilic conjugation is an alternative broad tissue agnostic delivery strategy^32,56,57^ that is now in clinical development for both local and systemic administration^33,58,59^ (ClinicalTrials.gov: NCT05881993, NCT06826196). Currently, several clinical trials are ongoing with both local administration for CSF/CNS and skin delivery as well as systemic administration for targeting placenta. For example, Comanche Biopharma reported a wide therapeutic index for sFLT1 targeting siRNAs conjugated to DCA class lipophilic conjugate in nonhuman primates for the treatment of preeclampsia. These constructs show systemic tolerability and high tissue exposure when injected subcutaneously (publicly presented preliminary data). These advances provide a foundation for the development of innovative treatments for diseases using lipophilic siRNAs. However, while these trials validate the use of hydrophobic siRNAs in the clinic more generally, if development were to pursue local muscular administration with these constructs in humans, diffusion and tissue coverage in a larger species would need to be assessed as these factors are critical for clinical translation.

Lastly, the therapeutic approach may also have implications in surgical management of established disease. At present, surgical excision of heterotopic bone remains the only option for restoring mobility in severe cases, yet recurrence following surgery is common due to trauma-induced re-ossification. Future studies should therefore investigate whether transient perioperative siRNA treatment could reduce postoperative lesion recurrence and expand the feasibility of surgical intervention in patients with advanced disease.

## Conclusions

In summary, this study provides the first proof of concept for the use of RNAi-based therapeutics in the treatment of FOP while limiting off-target adverse effects. We developed a combinatorial, flexible RNAi platform for the treatment of FOP, which may offer a path toward improved quality of life for patients affected by this devastating disease.

## Methods

### RNA Oligonucleotide Synthesis:

RNA oligonucleotides were synthesized by standard solid-phase phosphoramidite chemistry via the Dr. Oligo 48 for *in vitro* (Biolytic) or MerMade 12 for *in vivo* (BioAutomation). All oligos were synthesized with 2’-O-methyl and 2’-fluoro modifications (Chemgenes). *In vitro* sense strands were made at 1 μmol on cholesterol-conjugated support (Chemgenes); *in vivo* sense strands at 5 μmol on in-house DCA-functionalized CPG; antisense strands on Unylinker-CPG (Chemgenes). 5’-mono-phosphates were installed with a CED phosphoramidite (Chemgenes) for *in vitro* and a custom 5’-(E)-vinylphosphonate phosphoramidite for *in vivo* (Chemgenes); sense strands were 5’-Cy3 labeled (Gene Pharma).

### Oligonucleotide deprotection:

Sense strands were cleaved from CPG and deprotected in a 1:1 (v/v) mixture of 40% aqueous methylamine and 30% NH4OH at room temperature for 2 h. Antisense strands were cleaved and deprotected in 30% NH4OH with 3% diethylamine at 35°C for 20 h. After filtration to remove CPG, filtrates were frozen in liquid N2, SpeedVac-dried, and reconstituted in 5% acetonitrile for purification.

### HPLC purification:

Oligonucleotides were purified on an Agilent 1290 Infinity II HPLC. Sense strands were run on a Hamilton PRP-C18 reverse-phase column (buffer A: 50 mM sodium acetate in 5% acetonitrile; buffer B: acetonitrile) with a 0–20% (3 min) then 20–70% (23 min) gradient, 60°C and 40 mL/min. Antisense strands were purified on a SOURCE^™^ 15Q anion-exchange column (buffer A: 10 mM sodium acetate in 20% acetonitrile; buffer B: 1 M sodium perchlorate in 20% acetonitrile) using the same gradient and flow at 55°C. Elution was monitored at 260 nm, peak fractions were collected and confirmed by LC–MS, pooled, frozen, SpeedVac-dried overnight, reconstituted in water and desalted on Sephadex G-25.

### LC-MS analysis:

Oligonucleotide purity/identity were assessed by ion-pair RP LC–QTOF (Agilent 6530) on an Agilent 2.1×50 mm AdvanceBio C18 column (buffer A: 9 mM triethylamine/100 mM HFIP in H2O; buffer B: same in MeOH), 60°C, 0.5 mL/min, UV 260 nm. MS used ESI (negative mode), m/z 100–3200, 2 spectra/s, capillary 4000 V, fragmentor 180 V.

### Design of *ACVR1*^*R206H*^ SNP-selective siRNAs:

SNP-selective siRNAs were designed to target the human ACVR1R206H mutation (c.617G>A; p.R206H; NM_001111067.4) by generating consecutive guide strands spanning the SNP, identifying the optimal primary mismatch position, then introducing a secondary mismatch in guides flanking that site. Candidate siRNAs were excluded if they had >56% GC, runs of ≥4 identical nucleotides, complete homology to human miRNA seed sequences (antisense positions 2–7), or if antisense positions 2–17 were fully complementary to non-target transcripts. SiRNAs were named by mismatch positions (e.g., 6_9 = primary mismatch at position 6, secondary at 9), with cross-species names referenced to the human transcript.

### Design of *Il1b* siRNAs:

siRNAs were derived from 20-nt regions of human IL1B (NM_000576.3) and mouse Il1b (NM_008361) transcripts. Candidates (±10 nt upstream, +15 nt downstream) were scored by a weighted matrix and top hits per species selected; cross-species targeting required perfect homology across positions 2–17 (16 nt). siRNAs were named by transcript position (e.g., mouse siRNA 1293 = nt 1293–1312 of NM_008361), with cross-species names referenced to the human transcript.

### Cell culture and reagents:

HEL 92.1.7 (ATCC, #TIB-180) were maintained in RPMI-1640; HEK293T (ATCC, #CRL-3216), HeLa (ATCC, #CCL-2) and RAW 264.7 (ATCC, #TIB-71) were maintained in DMEM (Corning Cellgro, #10–013CV); all lines were cultured in complete media with 10% FBS (Gibco, #26140) and no antibiotics.

1) FOP and control iPSCs (from Dr. Edward Hsiao, UCSF) were ACVR1R206H-sequenced and maintained in primate ES cell medium (ReproCELL) on irradiated SNL feeders, with SNLs removed by ≥1 feeder-free passage before osteoblast differentiation.

2) FAPs were isolated from digested muscle of siRNA-treated, 8-week Acvr1^+/+^;Pdgfrα-GFP and Acvr1^R206H/+^;Pdgfrα-GFP mice using surface markers (GFP-Pdgfrα^+^Sca1^+^CD31^−^CD45^−^) and assessed by flow cytometry or FACS-sorted for RT-PCR/RNA-seq. BMSCs were isolated from 4-week Acvr1(R206H)Fl;Prrx1-Cre long bones and cultured in α-MEM (Gibco) with 10% FBS (Corning), 2 mM L-glutamine (Corning), 1% penicillin/streptomycin (Corning) and 1% NEAA (Corning), or differentiated in osteogenic medium with ascorbic acid (200 μM, Sigma, A8960) and β-glycerophosphate (10 mM, Sigma, #G9422).

3) Primary BMSCs were treated with LPS (InvivoGen, #LPS-B5), TNF (R&D, #210-TA), IL1B/IL1F2 (R&D, #201-LB) and Activin A (R&D, #338-AC). For mineralization, BMSCs were washed twice with PBS, fixed in 70% EtOH for 15 min RT, washed, stained with 2% Alizarin Red (Sigma, #A5533) for 5 min, washed 3× and quantified by acetic acid extraction (method60). For ALP, cells were fixed in 10% neutral buffered formalin and stained with Fast Blue (Sigma, #FBS25) and Naphthol AS-MX (Sigma, #855), or incubated with 1:10 Alamar Blue (Invitrogen, #DAL1100) for proliferation; ALP activity was measured after incubation with 6.5 mM Na2CO3, 18.5 mM NaHCO3, 2 mM MgCl2 and phosphatase substrate (Sigma, #S0942) using a spectrometer (BioRad).

4) Primary chondroprogenitors were isolated by dissecting knee joints of *Acvr1(R206H)Fl;Prrx1-Cre* neonates at postnatal day 0, removing soft tissues and skin, and excising femoral heads, condyles, and tibial plateaus. After overnight digestion with collagenase D, cartilage tissues were mechanically dissociated using sequentially smaller Pasteur pipettes and filtered through a 48 μm strainer. The cell pellet was resuspended in culture medium supplemented with 10% FBS and cultured under chondrogenic conditions. Chondrocytes were treated with siNTC or siACVR1 and either PBS or Activin A and 7 days later, cells were fixed with 4% paraformaldehyde and then stained with Alcian Blue solution (1% Alcian Blue, pH 1). The cells were destained with acetic acid solution for GAG quantification. Alternatively, total RNAs were subjected to RT-PCR to assess chondrogenic gene expression.

### *In vitro* passive uptake screening:

FOP iPSCs, HEL 92.1.7, and RAW 264.7 cells were treated with cholesterol-conjugated siRNAs targeting *ACVR1*^*R026H*^ and *Il1b* at a concentration of 1.5 μM, representing the maximal dose for dose-response assays. To induce expression of mouse *Il1b* mRNAs, RAW 264.7 cells were stimulated with LPS (1 μg/mL) for 8 hours after siRNA treatment, lysed with a diluted lysis mixture (Invitrogen, #13228) supplemented with 0.2 mg/mL of proteinase K (Invitrogen, #25530–049), and incubated at 55 °C for 30 min. Target mRNA levels were quantified using Quantigene 2.0 assays (Affymetrix) and normalized to mouse or human housekeeping gene *Hprt*. All QuantiGene detection probesets were purchased from ThermoFisher: human *IL1B* (#SB-10065), mouse *Il1b* (#SA-50396), human *HPRT* (#SA-10030), and mouse *Hprt* (#SA-15463).

### *In vitro* reporter screening assay:

HeLa and HEK293 cells were cultured in 3% fetal bovine serum (FBS) media, prepared by mixing 6% FBS media and Opti-MEM media in a 1:1 ratio and then, treated with cholesterol-conjugated siRNAs at a concentration of 1.5 μM, representing the maximal dose for dose-response assays. 3 days later, cells were transfected with 6 μg of reporter plasmids^35^ using Lipofectamine 3000 (Invitrogen, # L3000–015) according to the manufacturer’s protocol. 3 days later, cells were lysed and subjected for dual-luciferase assay (Promega, # E1960). *Renilla* activity was normalized to firefly luciferase activity, which was further normalized to untreated controls and plotted on a logarithmic scale.

### Mice:

Mice harboring a knock-in allele of human *ACVR1*^*R206H*^ (*ACVR1*^*(R206H)Fl*^) were obtained from the International FOP Association via Dr. Daniel Perrien (Emory University) and maintained on C57BL/6J background^63^. *ACVR1*^*(R206H)Fl*^ mice were crossed with *Sox2-Cre* mice where expression of Cre recombinase in epiblasts mediates expression of *ACVR1*^*R206H*^ in all tissues (*ACVR1*^*(R206H)Fl/+*^*;Sox2-cre,* hereafter referred to *Acvr1*^*R206H/+*^)^18^ or *Prrx1-Cre* mice where expression of Cre recombinase in Prrx1^+^ skeletal progenitors in the limb mesenchyme mediates *ACVR1*^*R206H*^-driven HO (*ACVR1*^*(R206H)Fl*^*;Prrx1-cre*). *ACVR1*^*R206H/+*^ mice were further crossed with *Pdgfrα-GFP* reporter mice to label Pdgfrα^+^ FAPs with GFP expression (*ACVR1*^*R206H/+*^*;Pdgfrα-GFP*). Alternatively, *ACVR1*^*R206H/+*^ mice were further crossed with *Il1r1*^*−/−*^ mice (Jackson laboratory, C57BL/6J) to delete *Il1r1* gene in FOP mice. Littermate controls were used for all experiments. All animals were used in accordance with the NIH Guide for the Care and Use of Laboratory Animals and were handled according to protocols approved by the University of Massachusetts Chan Medical School Institutional Animal Care and Use Committee (IACUC, 202200036).

**Table:** 

FOP mouse models used in this study		
*ACVR1*^*R206H/+*^ mice	*ACVR1* ^*(R206H)Fl/+*^ *;Sox2-cre*	Heterozygous expression of *ACVR1*^*R206H*^ in all tissues
*ACVR1*^*R206H/+*^*;Pdgfrα-GFP* mice	*ACVR1* ^*(R206H)Fl/+*^ *;Sox2-cre; Pdgfrα-GFP*	Homozygous expression of *ACVR1*^*R206H*^ in GFP-expressing PDGFR*α*^*+*^ FAPs
*ACVR1*^*R206H/+*^;*Il1r1*^*−/−*^ mice	*ACVR1*^*(R206H)Fl/+*^*;Sox2-cre*; *Il1r1*^*−/−*^	*ACVR1*^*R206H/+*^ and *IL-1R1* ^−/−^ expression in all tissues
*ACVR1*^*R206H*^ mice	*ACVR1* ^*(R206H)Fl*^ *;Prrx1-cre*	Homozygous expression of *ACVR1*^*R206H*^ in Prrx1^+^ skeletal progenitors

### *In vivo* efficacy test:

Individual tissues were harvested from siRNA-treated mice at the indicated time points, mechanically homogenized in 400 μL of homogenizing solution (Invitrogen; #QS0517) containing 0.2 mg/mL proteinase K (Invitrogen, #AM3546), and incubated at 55 °C for 30 min. Homogenized samples were then centrifuged at 14,000 × g for 5 min, and the clear supernatant was collected for subsequent analysis. The mRNA levels of human *ACVR1*^*R206H*^ and mouse *Il1b* were quantified using the QuantiGene Singleplex assay kit (Invitrogen; #QS0016) with human *ACVR1* (#SA-11235) and mouse *Il1b* (#SA-50396), respectively and normalized to mouse *Hprt* (#SA-15463).

### Peptide nucleic acid (PNA) hybridization assay:

Tissue biodistribution of siRNAs in mice was assessed using a PNA hybridization assay, as previously described^32,64^. Tissues were homogenized with proteinase K, incubated at 55°C for 30 min, then treated with 3 M KCl to remove SDS. antisense strands in the supernatant were hybridized to an Alexa 488-labeled probe complementary to the strand at 95°C, and the hybridized complexes were analyzed by anion-exchange HPLC with fluorescence detection (Agilent 1260, DNAPac PA100 column). Cy3 fluorescence peaks were quantified and compared to a calibration curve made from spiked tissues to determine siRNA concentrations.

### MicroCT and radiography:

Heterotopic ossification was assessed using a MicroCT 35 scanner (Scanco Medical) at 12 μm resolution. Whole limbs were excised, contoured around the tibia, and analyzed via 3D reconstructions from binarized images, with blinded investigators. The Inveon software was also used for fused 3D rendering. Representative images from genotypes (n>5) are shown. Whole-body radiographs were captured post-euthanasia using the Hologic Trident system at 1 mA and 28–30 kV with automatic exposure, with images from more than five animals.

### Histology, immunofluorescence, and immunohistochemistry:

Hind limbs were fixed in 10% formalin for 2 days, decalcified in 15% EDTA for 3 weeks, dehydrated, xylene-washed, infiltrated with paraffin, and sectioned at 7 μm. The sections were stained with Alcian Blue and counterstained with eosin to assess proteoglycan-rich cartilage formation. For frozen sections, tissues were fixed in 4% paraformaldehyde for 2–3 days, semi-decalcified with EDTA for 15 days at 4°C, infiltrated with 20% sucrose, embedded in OCT, and sectioned at 12 μm with a Cryostat. For immunohistochemistry, sections were subjected to antigen retrieval and blocking prior to incubation with antibodies specific to SOX9 (Cell Signaling Technology, #82630) and Aggrecan (Abcam, #ab216965). After incubation with appropriate secondary antibodies, signal detection was performed using a chromogenic substrate. Sections were counterstained with hematoxylin, dehydrated, and mounted for imaging.

### Intramuscular Efficacy in *Ex Vivo* Human Skin:

Freshly discarded post-orthopedic tumor resection quadriceps muscle was obtained from the Yale Biospecimen, Tissue, and Tumor Bank. Human specimens were rehydrated in phosphate-buffered saline (PBS), and adherent adipose tissue and connective tissue were carefully removed prior to processing. Tissue was then immersed in an antifungal solution diluted in PBS to minimize the risk of fungal contamination, followed by a PBS wash. siRNA was subsequently administered to the *ex vivo* muscle tissue and incubated in Iscove’s Modified Dulbecco’s Medium (Sigma-Aldrich; #I3390) supplemented with 20% FBS and 100 U/mL penicillin-streptomycin. For intramuscular delivery, 8 mm biopsy punches were taken from the muscle tissue, and 10 nmol of siRNA was administered via intramuscular injection in a total volume of 50 μL. Explants were maintained at 37°C for 96 hours in a 24-well plate format, with each well containing 2 mL of complete media. Target gene mRNA expression was quantified using the QuantiGene 2.0 assay.

### Complete blood count (CBC) test:

CBC tests were performed to evaluate cellular components in the blood of siRNA-treated mice, including white blood cells (WBCs), red blood cells (RBCs), lymphocytes, monocytes, neutrophils, hemoglobulin (HG), and platelets (PLTs). Blood drops were collected into a microtainer EDTA tube and tested within 1 hr at room temperature using an automated hematology analyzer (VetScan HM5, Zoetis.USA).

### Quantitative RT-PCR, RT^2^ profiler PCR arrays, and immunoblotting:

Total RNA was extracted from cells and tissues using QIAzol (QIAGEN), then reverse-transcribed into cDNA with the High-Capacity cDNA Reverse Transcription Kit (Applied Biosystems, #4368814). qRT-PCR was performed with iTaq^™^ Universal SYBR^®^ Green Supermix (Bio-Rad, #1725122) using a CFX connect RT-PCR detection system (Bio-Rad); for injured muscle samples, tissues were snap-frozen in liquid nitrogen for 30 sec and homogenized in 1 ml of QIAzol for 1 min. Alternatively, human iPSCs, mouse BMSCs, and FACS-sorted FAPs were lysed in QIAzol and used for RT-PCR analysis (primers listed in Extended Data Table 4). RT2 profiler PCR arrays (QIAGEN) measured mRNA levels of 84 inflammatory cytokines and receptors (see Extended Data Table 5). Cells were lysed in TNT lysis buffer (150 mM NaCl, 1% Triton X-100, 1 mM EDTA, 1 mM EGTA, 50 mM NaF, 1 mM Na3VO4, 1 mM PMSF, and protease inhibitor cocktail from Sigma). Protein concentrations were measured using the DC protein assay (Bio-Rad), and equivalent amounts were subjected to SDS-PAGE, transferred to Immobilon-P membranes (Millipore), and immunoblotted with anti-ACVR1 (1:1000, Sigma, #SAB3500435), anti-GAPDH (1:1000, EMD Millipore, #CB1001), anti-phospho-SMAD1/5 (1:1000, Cell Signaling Technology, #9516), anti-phospho-ERK1/2 (1:1000, Cell Signaling Technology, #9101), anti-ERK1/2 (1:1000, Cell Signaling Technology, #9102), and anti-HSP90 (1:1000, BioLegend, #675402). Blots were developed with ECL (ThermoFisher Scientific); anti-HSP90 or anti-GAPDH served as loading controls.

### Sanger sequencing and Next-generation sequencing (NGS):

Human FOP iPSCs were treated with 1 μM of siRNA (NTC or SNP6.9) for 3 days. The cDNAs synthesized from total RNA were amplified using ACVR1-targeting primers and the PCR products was subjected for Sanger Sequencing or Next-Generation Sequencing (NGS). Transcripts of *ACVR1*^*WT*^ and *ACVR1*^*R206H*^ in siRNA-treated cells were quantitated by PCR amplicons using NGS in the Massachusetts General Hospital Center for Computational & Integrative Biology DNA Core (Boston, MA).

### RNA sequencing and whole transcriptome analysis:

Trauma-injured tibial muscles from siRNA-treated *ACVR1*^*+/+*^*;Pdgfrα-GFP* and *ACVR1*^*R206H/+*^*;Pdgfrα-GFP* mice were minced, digested in 0.2% Collagenase Type II and 2.4 U/mL Dispase II at 37°C for 60 min, then filtered through a 70 μm strainer to obtain single cells. The cells were stained with anti-CD45 and anti-CD31 (negative selection) and anti-Sca1 and GFP (positive selection for GFP+ FAPs), then sorted via BD FACSDiva 8.0.1. RNA was extracted immediately using the PureLink RNA Mini Kit (Invitrogen, #12183018A), and RNA integrity was confirmed with Agilent Bioanalyzer 2100, selecting samples with RIN > 9.0 for RNA-seq. Sequencing was performed by Innomics INC. on the DNBSEQ platform (paired-end 150 bp). Raw read quality was checked with FASTQC (v0.11.8). Reads aligning to rRNA/tRNA were filtered using Bowtie2 or STAR. Reads were aligned to the mm10 genome with HISAT2, and gene/isoform expression quantified with RSEM. Differential expression analysis was conducted using DESeq2 with ‘ashr’ for LFC shrinkage; significant DEGs were identified with FDR < 0.05 and |LFC| > 0.585. GSEA was performed using GSEA^65^.

### Flow cytometry analysis of FOP-relevant cell populations in HO lesions:

Trauma-injured tibial muscles from siRNA-treated *ACVR1*^*+/+*^*;Pdgfrα-GFP* and *ACVR1*^*R206H/+*^*;Pdgfrα-GFP* mice were minced, digested in 0.2% Collagenase Type II and 2.4 U/mL Dispase II at 37°C for 60 min, then filtered through a 70 μm strainer before resuspending into FACS buffer and then incubated with Fc blocking buffer (BD Biosciences, #564765) for 15 min at 4°C. After Fc receptor blocking, cells were treated with fluorochrome labelled antibody cocktail including anti-CD45, anti-CD31, anti-Sca1, anti-CD11b (1:100, BioLegend, #101212), anti-CD45R (B220) (1:100, Tonbo, #65–0452), anti-CD3ε (1:100, BioLegend, #100203), anti-CD11c (1:100, BioLegend, #117337), anti-Ly6G (1:100, BioLegend, #127618), anti-Ly6C (1:100, BioLegend, #128006), anti-Gr-1 (1:100, Invitrogen, #11–5931-85), anti-CXCR4 (1:100, BioLegend, #146507) and anti-integrin β−1 (1:100, BioLegend, #102205) in cold FACS buffer. After treatment with Ghost Dye red 780 (1:1000, Tonbo, #13–0865-T100) for live/dead cell discrimination, cells were then subjected to acquisition on a BD LSR II flow cytometer (BD Biosciences). The data were analyzed using FlowJo (v.10.1).

### Statistical analysis and reproducibility:

All experiments (histology, microCT, X-ray, RT-PCR, bDNA, immunofluorescence, IHC, immunoblotting) were conducted with ≥3 biological replicates, repeated 2–3 times for reproducibility. Data are shown as mean ± SD. Statistical analyses were performed using GraphPad Prism (v10.4.0). Normality was tested with the Shapiro-Wilk test and confirmed with QQ plots. If p > 0.05, two-tailed unpaired Student’s t-test was used; if p ≤ 0.05, Mann-Whitney U test was applied. For ≥ 3 groups, one-way ANOVA was used if normality was met; variances were assessed with the Brown-Forsythe test. Unequal variances (p ≤ 0.05) led to Welch’s ANOVA, followed by Dunnett’s T3. Significance was set at p < 0.05.

## Supplementary Files

This is a list of supplementary files associated with this preprint. Click to download.

- YangetalSuppinfo.pdf

## Data Availability

Data supporting the findings of this manuscript are available from the corresponding authors upon request. The raw data are protected and are not available due to data privacy laws. The minimum dataset generated in this study necessary to interpret, verify and extend the research in the article are provided in the Supplementary Information/Source Data file.
